# Green Synthesis of Silver Nanoparticles Derived from *Papaver rhoeas* L. Leaf Extract: Cytotoxic and Antimicrobial Properties

**DOI:** 10.3390/molecules28176424

**Published:** 2023-09-04

**Authors:** Polat İpek, Reşit Yıldız, Mehmet Fırat Baran, Abdulkerim Hatipoğlu, Ayşe Baran, Albert Sufianov, Ozal Beylerli

**Affiliations:** 1 Department of Physiology, Faculty of Veterinary Medicine, Dicle University, Diyarbakir 21280, Turkey; polat.ipek@dicle.edu.tr; 2 Department of Nutrition and Dietetics, Faculty of Health Sciences, Mardin Artuklu University, Mardin 47200, Turkey; ryildiz80@gmail.com; 3 Department of Food Processing, Vocational School of Technical Sciences, Batman University, Batman 72060, Turkey; 4 Department of Biology, Graduate Education Institute, Mardin Artuklu University, Mardin 47200, Turkey; ayse.gorgec43@gmail.com; 5 Educational and Scientific Institute of Neurosurgery, Peoples’ Friendship University of Russia (RUDN University), Moscow 117198, Russia; sufianov@gmail.com; 6 Department of Neurosurgery, Sechenov First Moscow State Medical University (Sechenov University), Moscow 119435, Russia; 7 Central Research Laboratory, Bashkir State Medical University, Ufa 450008, Russia

**Keywords:** anticancer, green synthesis, pathogen microorganisms, silver nanoparticles, TEM, XRD

## Abstract

In the last few decades, the search for metal nanoparticles as an alternative to cancer treatments and antibiotics has increased. In this article, the spectroscopic (ultraviolet–visible (UV-vis), electron-dispersing X-ray (EDX), and Fourier transform infrared (FT-IR)), microscopic (field emission scanning electron microscope (FE-SEM), transmission electron microscope (TEM), and atomic force microscope (AFM)), structural (X-ray diffractometer (XRD) and zetasizer), and analytic (thermogravimetric/differential thermal analyzer (TGA-DTA)) characterization of the silver nanoparticles (AgNPs) produced from *Papaver rhoeas* (PR) L. leaf extract are presented. PR-AgNPs are generally spherical and have a maximum surface plasmon resonance of 464.03 nm. The dimensions of the manufactured nanomaterial are in the range of 1.47–7.31 nm. PR-AgNPs have high thermal stability and a zeta potential of −36.1 mV. The minimum inhibitory concentration (MIC) values (mg L^−1^) of PR-AgNPs on *Staphylococcus aureus*, *Escherichia coli*, *Bacillus subtilis*, *Pseudomonas aeruginosa*, and *Candida albicans* are 1.50, 0.75, 3.00, 6.00, and 0.37, respectively. In the study, the cytotoxic and proliferative effects of PR-AgNPs using the MTT (3-(4,5-dimethylthiazol-2-yl)-diphenyltetrazolium bromide) method on various cancer cell lines (CACO-2 (human colon adenocarcinoma cell), MCF-7 (human breast cancer cell), T98-G (glioblastoma multiforme cell), and healthy HUVEC (human umbilical vein endothelial cell)) cell lines are presented. After 24 and 48 h of the application, the half-maximum inhibitory concentration (IC_50_) values (μg mL^−1^) of PR-AgNPs on HUVEC, CACO-2, MCF-7, and T98-G lines are 2.365 and 2.380; 2.526 and 2.521; 3.274 and 3.318; 3.472 and 3.526, respectively. Comprehensive in vivo research of PR-AgNPs is proposed to reveal their potential for usage in sectors such as nanomedicine and nanochemistry.

## 1. Introduction

The World Health Organization (WHO) reports that cancer, one of the leading causes of death worldwide, caused the death of approximately 10 million people in 2020. Cancer is a deadly disease that involves the irregular growth of cells. In the last quarter century, scientists have started to use metal nanoparticles, which have the unique ability to better target gene silencing, drug delivery, and sensing an alternative to conventional techniques for biomedical and environmental applications [1,2,3]. In this context, the synthesis of metal nanoparticles using green chemistry procedures is especially preferred, called “green nanotechnology”, which does not contain toxic reducing and stabilizing substances [4,5,6,7]. The development of nanotechnology and green nanoparticles has made positive contributions to the solution of problems affecting human health on a global scale, such as cancer studies, alternative antibiotic applications, and infectious diseases [8,9].

The poppy (*Papaver rhoeas* L.) (*P. rhoeas*, PR) plant is one of the most commonly utilized plants in the medicinal area due to its abundance in numerous active phytochemical elements, particularly phenolic, as well as other compounds [8]. *P. rhoeas*, an annual plant, is one of the 200 species belonging to the Papaveraceae family. *P. rhoeas* is a plant with red flowers and whitish hairs, leaves of various shapes and sizes, whose height is usually around 20–80 cm. The kidney-like seeds of *P. rhoeas* can remain dormant for many years when placed in the soil. It is a widespread weed plant native to Europe, North Africa, and Western Asia particularly in the Eastern Mediterranean region; however, its actual origin is unknown. *P. rhoeas*, which has a widespread distribution in Turkey, is utilized in folk medicine as cough syrup, depressant (sedative), pain reliever, diarrhea treatment, skin irritation treatment, and insomnia tea for children. Fresh petals, seeds, and flowers are used as food for human nutrition, especially in the southwestern region of Turkey [8].

Previous studies showed that the four parts of *P. rhoeas* have notable antibacterial and antioxidant properties [10]. The stem has the highest antimicrobial activity, while the leaves and flowers have the highest antioxidant activity [10]. Furthermore, silver nanoparticles (AgNPs) derived from bio-reduction with *P. rhoeas* significantly inhibited the growth ability of bacterial species such as *P. aeruginosa*, *S. enterica*, and *K. pneumoniae* showing the highest activity in this regard [9].

Surface modification of metal nanoparticles is preferred because they do not interact with other elements and can be adjusted to nanoscale length by controlling parameters such as the modification, size, and shape of metal for antibacterial applications [11].

AgNPs have been extensively utilized in anticancer, antibacterial, anticandidal, and antiviral studies due to their strong inhibition effects on cell proliferation [12]. AgNPs adhere to the cell wall, impairing the permeability of the cell wall and cellular respiration. AgNPs can also cause cellular devastation by acting on the cell’s DNA and compounds containing phosphorus and sulfur such as protein [13]. The superior properties of AgNPs have also made them useful in different fields such as agriculture, water treatment, drug delivery, data storage, cosmetics, textiles, cell biology, chemical sensing, and the food sector [14].

This research was carried out to reveal the anticancer and antipathogenic capacity of AgNPs produced by the *P. rhoeas* plant. For this purpose, AgNPs were synthesized from *P. rhoeas* leaf extract and silver nitrate (AgNO_3_) aqueous solution. After the spectroscopic, microscopic, and analytical characterization of PR-AgNPs, their cytotoxic, antibacterial, and anticandidal activities were investigated.

## 2. Results and Discussion

### 2.1. Ultraviolet–Visible Spectroscopy Analysis

**Figure 1:**
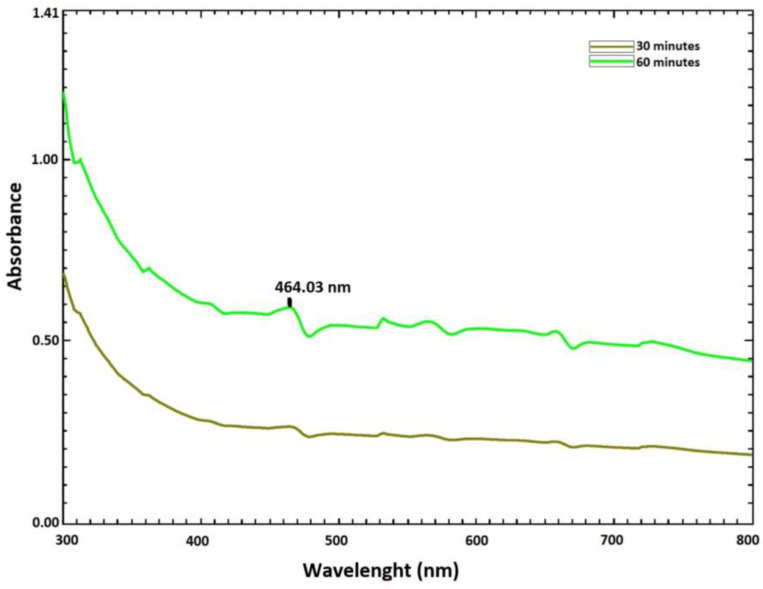
Ultraviolet–visible spectrum data of *Papaver rhoeas* leaf extract-based silver nanoparticles.

In an aqueous solution, the color of the AgNPs produced by the biosynthesis of *P. rhoeas* leaf extract changed from yellow to black. As is known, UV-Vis spectroscopy can be used to examine size- and shape-controlled nanoparticles in aqueous suspensions [15]. The samples were scanned at different time intervals (30 and 60 min) between 300 and 800 nm using UV-Vis spectrophotometer to detect the maximum wavelength. The maximum wavelength of PR-AgNPs, determined as 464.03 nm, is caused by the surface plasmon resonance obtained from the excitation of free electrons in the silver metal during synthesis (Figure 1). As is known, the optical properties of AgNO_3_ solution change when exposed to phytochemicals in the plant extract due to a decrease in the silver ions first to elemental silver and then to AgNPs [16].

### 2.2. Field Emission Scanning Electron Microscopy and Transmission Electron Microscopy Analysis

**Figure 2:**
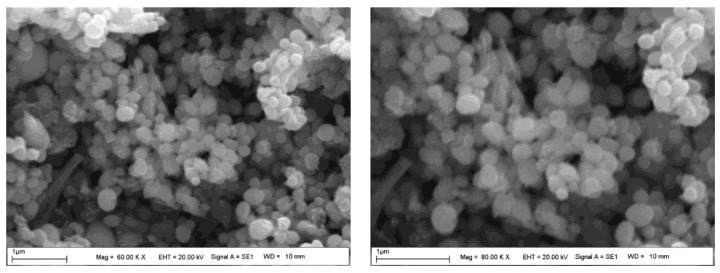
Field emission scanning microscopy pictures (60 KX and 80 KX) of *Papaver rhoeas* leaf extract-based silver nanoparticles.

**Figure 3:**
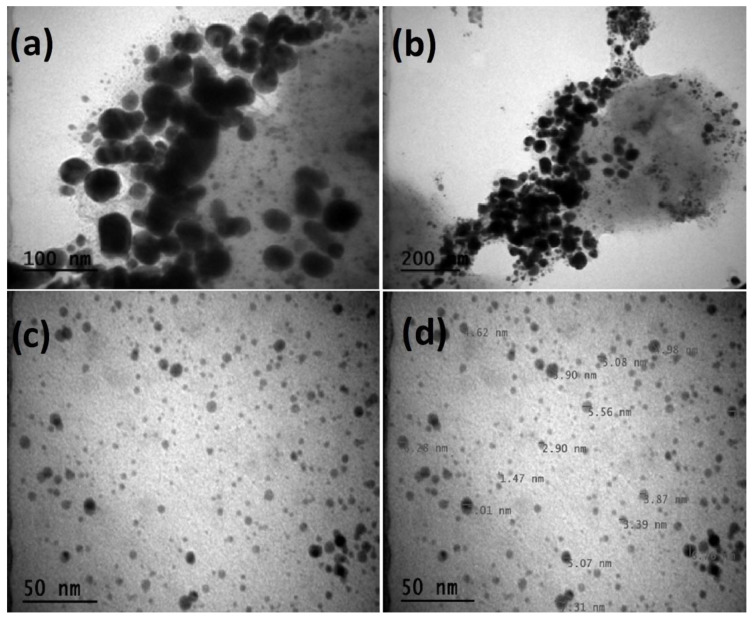
Transmission electron microscopy images of *Papaver rhoeas* leaf extract-based silver nanoparticles ((**a**): 100 nm, (**b**): 200 nm, (**c**): 50 nm, and (**d**): 50 nm (measured)).

FE-SEM and TEM micrograph images (Figure 2 and Figure 3a–d) of AgNPs synthesized from *P. rhoeas* leaf extract indicate the formation of single spherical particles.

To detect the morphological structure, dimension, and multidistribution of the biogenically synthesized AgNPs in more detail, TEM images were taken at 50 nm, 100 nm, and 200 nm scales (Figure 3a–d). According to the TEM findings, the dimensions of the manufactured nanomaterial were in the range of 1.47–7.31 nm (Figure 3d). On the other hand, it was observed that the produced nanomaterial was mostly spherical and coexisted in durable tiny communities due to useful phytochemicals and surface charges (Figure 3a–d) [17]. Numerous researchers also reported plant-based spherical-shaped nanoparticles [18,19]. AgNPs with this shape readily penetrate past the cell membrane, can injure objective cell organelles and enzymatic procedures, and cause injured cells to undergo apoptosis [20].

### 2.3. Electron Dispersive X-ray Spectroscopy Analysis

**Figure 4:**
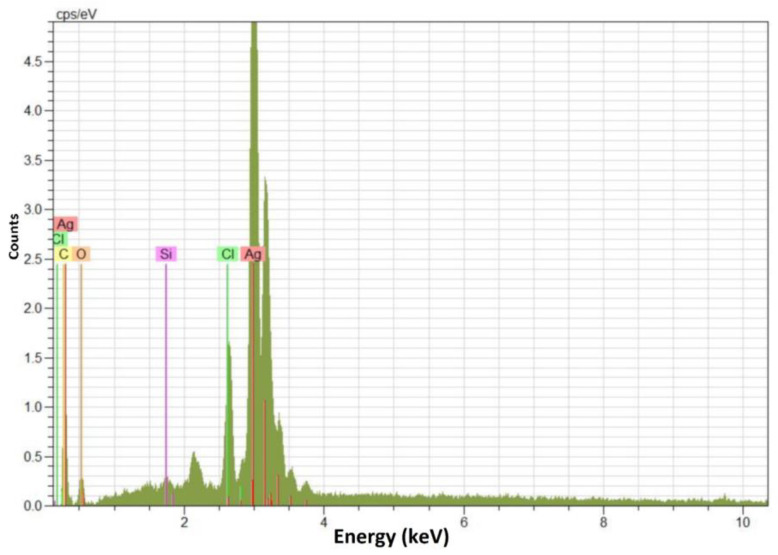
Electron dispersive X-ray spectrum of *Papaver rhoeas* leaf extract-based silver nanoparticles.

The EDX spectrum of PR-AgNPs (Figure 4) showed the characteristic optical absorption cap at about 3 KeV, decisive for metallic AgNPs [21]. EDX analysis revealed the proportion of AgNPs to be 79.41%. In addition, considering the weak signals from the pollutants in the EDX spectrum, oxygen (O), carbon (C), chlorine (Cl), and silicon (Si) were detected as 10.93%, 8.82%, 0.62%, and 0.22%, respectively. Despite being thoroughly cleansed with pure water, the produced nanomaterial may still contain certain pollutants.

### 2.4. X-ray Diffraction Analysis

**Figure 5:**
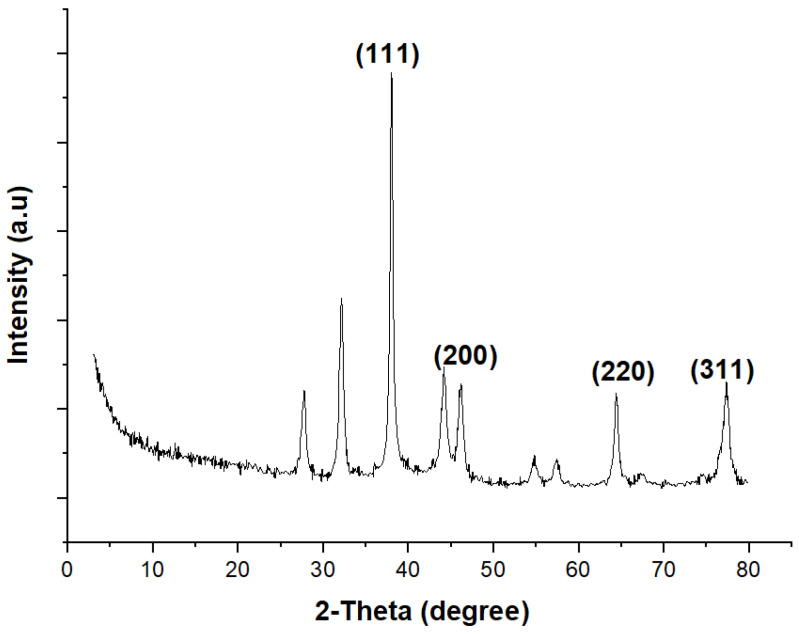
X-ray diffraction result of *Papaver rhoeas* leaf extract-based silver nanoparticles.

XRD measurements validated the crystal structure of PR-AgNPs (Figure 5). In the XRD spectrum model, the diffraction summits at 38.25°, 44.59°, 64.78°, and 77.56°, which indicate the face-centered cubic crystal form of silver at 2θ = 38.25, correspond to the planes (111), (200), (220), and (311), respectively. Some small peaks in the XRD pattern are due to phytochemicals or pollution involved in the reduction. Many plant-based AgNPs synthesis experiments produced peaks resembling the crystal structure of silver, including *Thymbra spicata* [22] and *Ananas comosus* [16]. The cubic structure of AgNPs was calculated from the Debye Scherrer equation (D = Kλ/βCosθ). The mean crystal dimension of PR-AgNPs was reckoned as approximately 17.77 nm, with 38.25 being the highest peak in the XRD pattern.

### 2.5. Fourier Transform Infrared Spectroscopy Analysis

**Figure 6:**
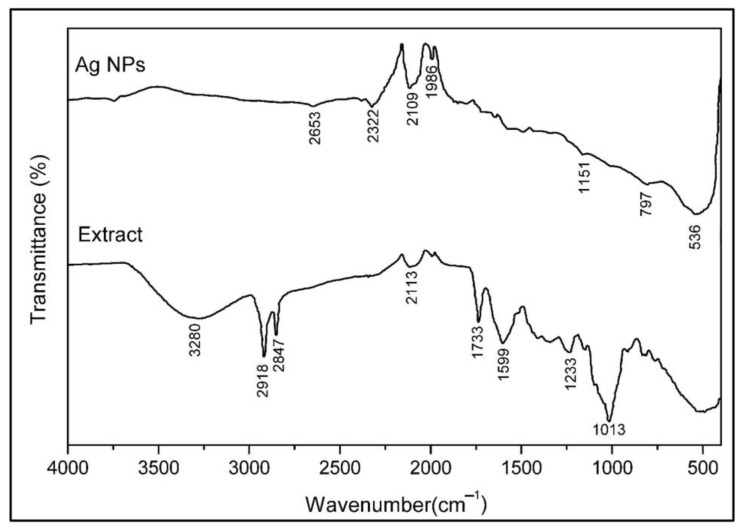
Fourier transform infrared (FT-IR) spectra of *Papaver rhoeas* leaf aqueous extract and biosynthesized AgNPs.

The FT-IR spectrum was used to explain the presence of some functional groups in the plant extract, possible biomolecules in the extract, and biomolecules that prominently bind to the surface of the AgNPs, as shown in Figure 6. The FT-IR spectrum of the plant extract showed peaks at 3280, 2918, 2847, 2113, 1733, 1599, 1233, and 1013 cm^−1^. In contrast, PR-AgNPs showed peaks at 3736, 2653, 2322, 2019, 1986, 1151, 797, and 536 cm^−1^. The broad peak at 3280 cm^−1^ is attributed to the -OH stretch of alcohol or phenol [23]. Significant peaks at 2918, 2847, and 2653 cm^−1^ are expressed as C–H (alkaline) stress peaks [24]. The peak at 2322 cm^−1^ may also belong to the isocyanate and phosphate groups. The peaks (-C≡N or -C≡C-) at 2113 and 2109 cm^−1^ correspond to the tensile vibration [19]. It is thought that the functional groups corresponding to the peak at 1599 cm^−1^ are N=O and C=C. Furthermore, 1733, 1599, 1233, 1148, and 1013 cm^−1^ may indicate the presence of ester, amide, amine, and phenol groups [9]. The peak vibration at 1599 cm^−1^ corresponding to the C–C carbon bond causes broadening. The peaks present at 1233 and 1148 cm^−1^ indicate the presence of C–N amine groups and C=O carboxylic groups, respectively [25]. The 1151 and 1013 cm^−1^ peaks at 797 and 536 cm^−1^ (OCN deformation) indicate the presence of C–H bending caused by heterocyclic mixtures of flavonoids [26].

When the *P. rhoeas* FT-IR pattern is compared with the solid powdered PR-AgNPs formed as a result of the reaction, it is seen that the prominent functional groups in the plant are lost, or their intensity is reduced (Figure 6).

### 2.6. Thermogravimetric Analysis–Differential Thermal Analysis

**Figure 7:**
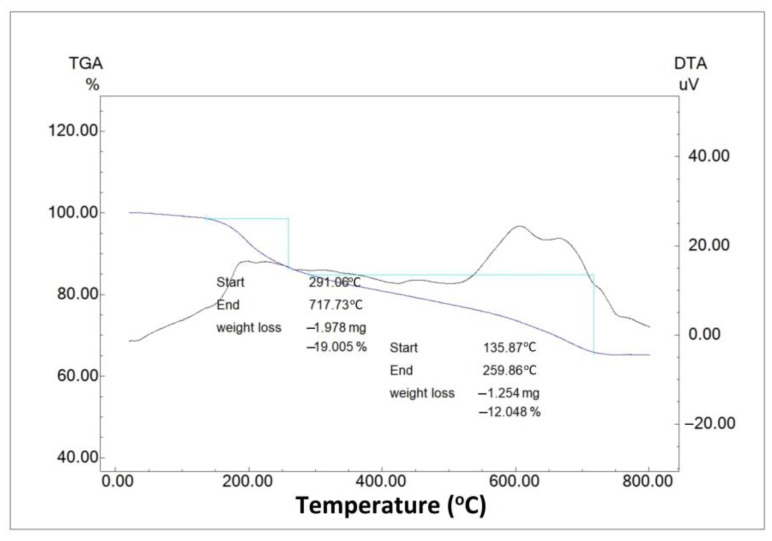
Thermogravimetric/differential thermal analyzer results of *Papaver rhoeas* leaf extract-based silver nanoparticles.

The heat resistance of the produced AgNPs, the weight loss during heating, and the temperature changes induced by exothermic/endothermic reactions were all determined via TGA-DTA analysis (Figure 7). As a result, the total quantity of phytochemical residues on the surface of PR-AgNPs was evaluated [27]. According to the findings, approximately 12% mass loss occurred in the sample at 135.87–259.86 °C (Figure 7). This loss can be due to the degradation of flavonoids, phenolic acids, and carbohydrates resulting from the plant leaf extract and due to being in charge of the stabilization of the AgNPs [28]. On the other hand, approximately 19% mass loss occurred in the sample at temperatures between 291.06 and 717.73 °C (Figure 7). The loss at these high temperatures can be considered as the breakdown of refractory aromatic compounds attached to the outside of the AgNPs [29]. As can be seen, the mass loss is greater at very high temperatures. In other words, the nanomaterial diminishes gradually, and the AgNPs produced are robust and resilient even at extreme temperatures.

### 2.7. Atomic Force Microscopy Analysis

**Figure 8:**
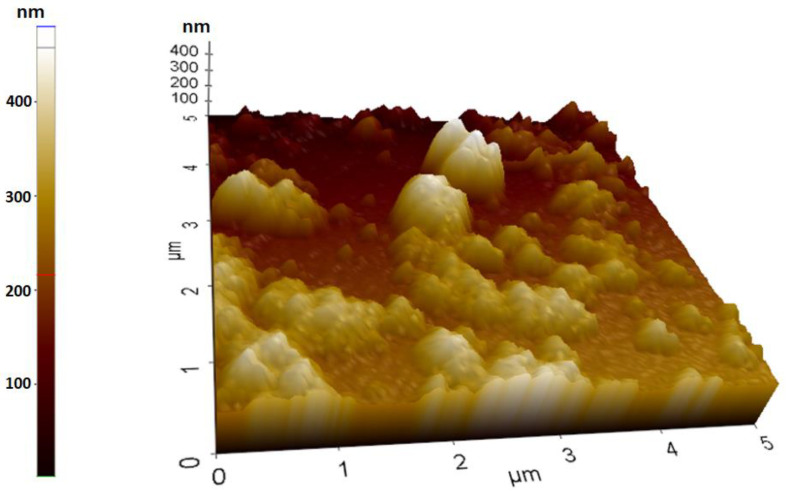
Atomic force microscopy of *Papaver rhoeas* leaf extract-based silver nanoparticles.

The AFM was utilized to characterize the two- and three-dimensional (2D-3D) topographic characteristics and surface roughness of the AgNPs (Figure 8). AFM is applied as a structural characterization technique for the study of silver nanomaterials in contact mode. The 2D dimensional AFM image taken at a 1 nanometer scale shows the homogeneous distribution of mostly spherical PR-AgNPs without agglomeration (Figure 8). That is to say, the AFM findings agree with those of the FE-SEM and the TEM.

### 2.8. Antimicrobial Activity

Minimum inhibitory concentration values allow us to obtain satisfactory ideas about the potency of AgNPs against different pathogenic microorganisms. The results of in vitro effects of PR-AgNPs synthesized in aqueous media against pathogenic yeast and bacteria are demonstrated in Table 1. According to the obtained MIC outcomes, PR-AgNPs suppressed the growth of *C. albicans* more effectively than AgNO_3_ and antibiotics. The inhibition effects of PR-AgNPs on pathogens were *C. albicans* > *E. coli* > *S. aureus* > *P. aeruginosa* > *B. subtilis*.

The strong inhibitory effect of the biogenic AgNPs on *C. albicans* was also supported by different studies [9,30]. However, contrary to this situation, some researchers reported that AgNPs were mostly more effective on bacteria than fungi [31].

Silver is more poisonous to microorganisms but less hazardous to mammalian cells than the other metals. But, its effect is short-lived due to the disadvantage of the complex formation of silver ions. On the other hand, AgNPs in inert form, which act as an antibiotic by triggering the manufacturing of reactive oxygen species (ROS), eliminated the disadvantage [32]. Moreover, the fungicidal mechanism of AgNPs against fungi has not been sufficiently elucidated so far [33].

### 2.9. Zeta Analysis

**Figure 9:**
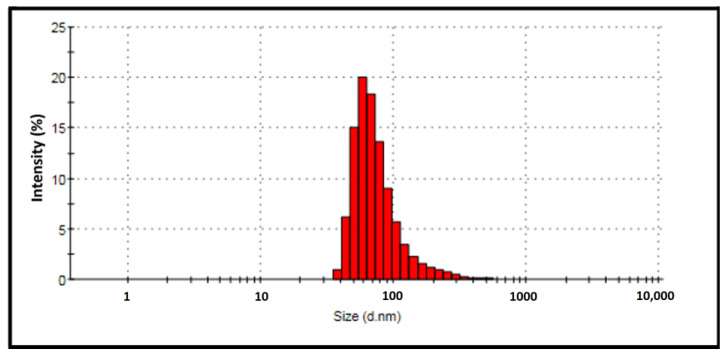
The size distribution density of *Papaver rhoeas* leaf extract-based silver nanoparticles.

**Figure 10:**
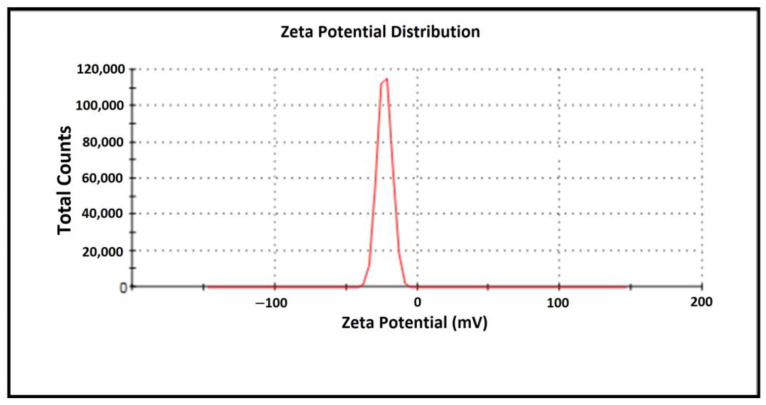
Zeta potential of *Papaver rhoeas* leaf extract-based silver nanoparticles.

The zeta size of biogenic AgNPs ranges between 1 and 150 nm, as seen in Figure 9. The zeta potential for the nanomaterial was determined to be −36.1 mV (Figure 10). The structure’s absorption of OH^−^ ions causes a negative zeta potential value. These OH^−^ ions prevent aggregation and ensure a stable and uniform distribution of the synthesized nanomaterial [34,35]. Several researchers found negative zeta potential values of AgNPs produced from diverse plants, which supports the current work [34,36].

### 2.10. Cytotoxic Effects

**Figure 11:**
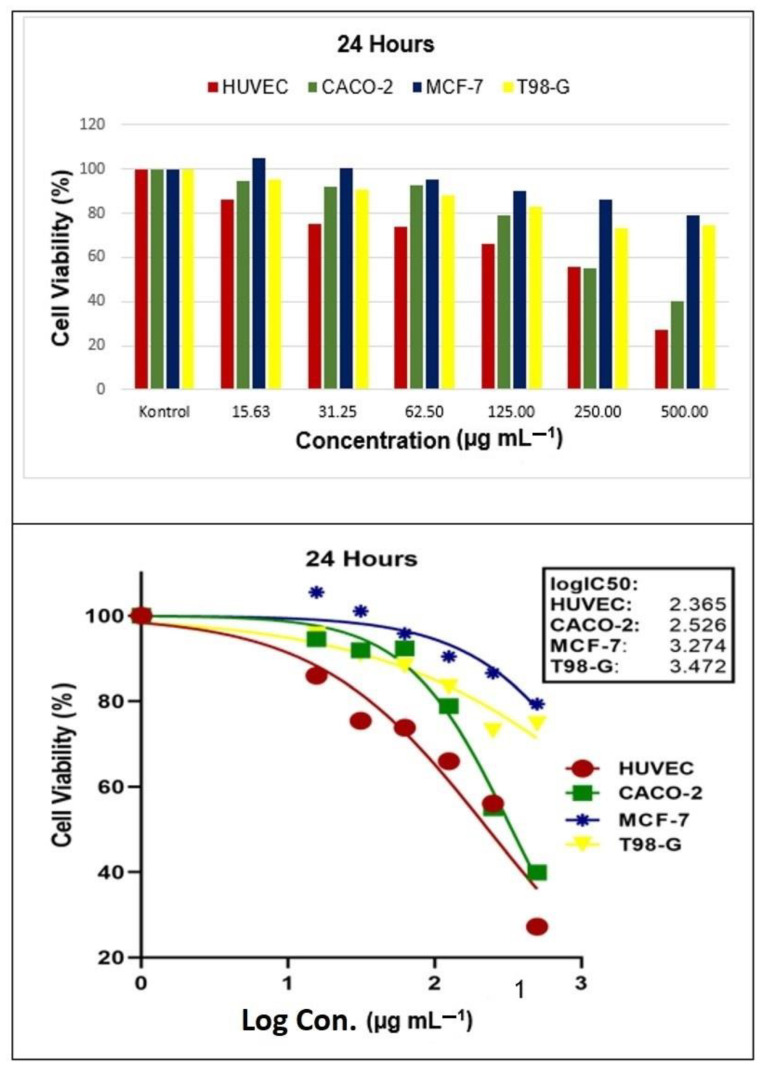
The half maximum inhibitory concentration (IC_50_) results of *Papaver rhoeas* leaf extract-based silver nanoparticles on human colon adenocarcinoma cell (HUVEC), human breast cancer cell (CACO-2), glioblastoma multiforme cell (MCF-7), and healthy human umbilical vein endothelial cell (T98-G) lines at 24 h.

**Figure 12:**
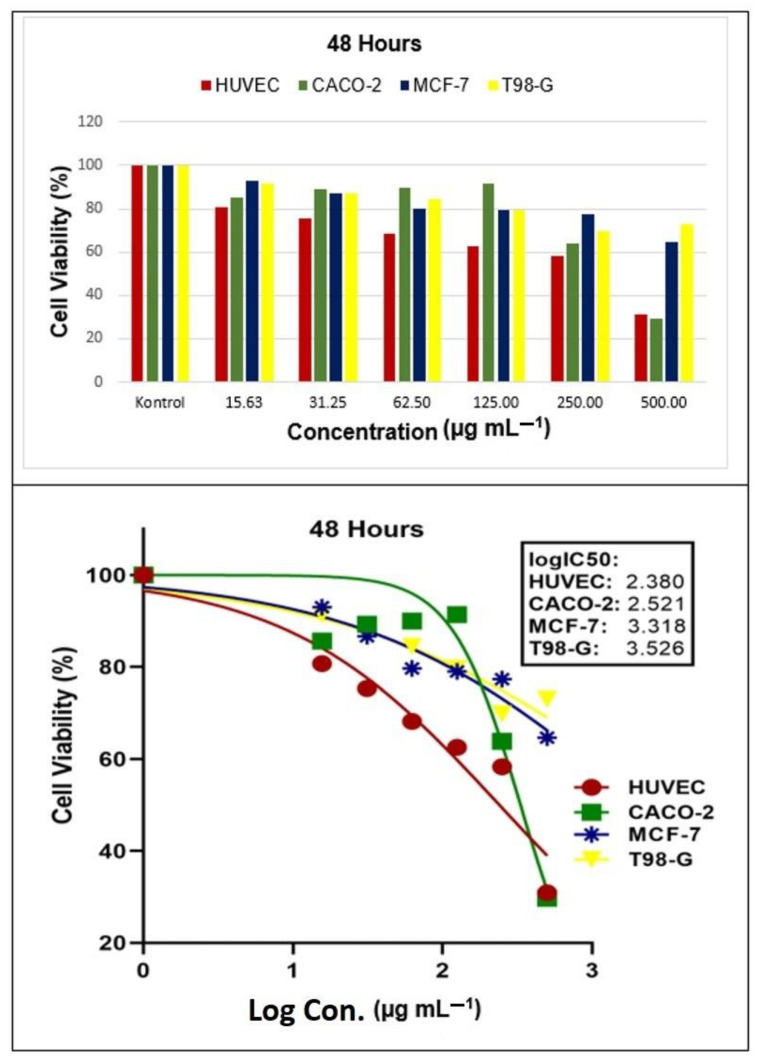
The half maximum inhibitory concentration (IC_50_) results of *Papaver rhoeas* leaf extract-based silver nanoparticles on human colon adenocarcinoma cell (HUVEC), human breast cancer cell (CACO-2), glioblastoma multiforme cell (MCF-7), and healthy human umbilical vein endothelial cell (T98-G) lines at 48 h.

After applying the AgNPs, it was discovered that it could prevent the growth of HUVEC (healthy human umbilical vein endothelial cell), CACO-2 (human colon adenocarcinoma cell), MCF-7 (human breast cancer cell), and T98-G (glioblastoma multiforme cell) lines (Figure 11 and Figure 12). Upon examination of the results, it was noticed that the rate of cell death increased significantly as the concentration increased during both time studies. However, when comparing the two-time studies, it was found that the cell viability rates were not statistically significant. In other words, the deaths depended on the dosage but not the time.

After 24 and 48 h of the application, the IC_50_ values (µg mL^−1^) on HUVEC, CACO-2, MCF-7, and T98-G cells were as follows: 2.365 and 2.380; 2.526 and 2.521; 3.274 and 3.318; 3.472 and 3.526, respectively.

Several independent studies have shown that biogenic AgNPs (µg mL^−1^) have antiproliferative effects ranging from 5.12 to 58.00 for MCF-7 [37,38] and 5.00 to 90.00 for CACO-2 [38,39]. The IC_50_ levels of cancer cell lines used in this study were lower than those reported by other researchers. Additionally, AgNPs have been found to have high antiproliferative effects against various cancer cell lines, including HCT-116 (colon carcinoma cell) and HepG2 (hepatocellular carcinoma) [40]. It is believed that the cytotoxic effect levels on the cells are determined by the morphological structure and size of AgNPs synthesized in the studies.

## 3. Materials and Methods

### 3.1. Plant Samples, Chemicals, and Microorganisms

*P. rhoeas* leaves obtained from the southeast of Turkey (Kilis-Alatepe, 79002) were utilized in the reduction reactions of AgNO_3_ salt. *P*. *rhoeas* GPS location: 36°47′13″ N, 37°11′57″ D. 36°47′13″ N, and 37°11′57″ D on the GPS.

The antibiotics used in the study (colistin for Gram-positive, fluconazole for yeast, and vancomycin for Gram-negative) and AgNO_3_ (99.8 purity) were bought from Sigma-Aldrich (Darmstadt, Germany). Pathogenic *Staphylococcus aureus* (ATCC^®^ 29213™), *Pseudomonas aeruginosa* (ATCC^®^ 27853™), *Bacillus subtilis* (ATCC^®^ 11774™), *Escherichia coli* (ATCC^®^ 25922™), and *Candida albicans* (ATCC^®^ 10231™) were used to reckon the growth suppressive impact of generated AgNPs.

### 3.2. Plant Leaf Extract Preparation

Deionized water was used to properly clean fresh *P. rhoeas* leaves before they were dried at 40 °C. A total of 25 g of dry leaves was mixed with 250 mL of distilled water, boiled at 90 °C, and then cooled. It was then filtered using filter paper. The extracted material was cooled to ambient temperature. To be utilized in the biosynthesis of AgNPs, it was then stored in a cold environment (+4 °C).

### 3.3. Manufacturing of Silver Nanoparticles from Plant Leaf Extract

To accomplish the green production of AgNPs, a 40 mM AgNO_3_ aqueous solution was provided utilizing solid AgNO_3_. A reaction between 200 mL of *P. rhoeas* leaf extract and 200 mL of AgNO_3_ solution was allowed to occur at 50 °C. The solution was centrifuged for the specified duration and velocity (4800 rpm, 20 min). The solid phase accumulated at the bottom was rinsed several times with distilled water. The resultant solid was dried in a furnace on a watch glass for 1 day at 55 °C. The dried nanomaterial was preserved for characterization and biological applications.

### 3.4. Structural Characterization and Thermal Properties of Silver Nanoparticles

Ultraviolet–visible (UV-vis.) spectroscopy (Agilent, CARY 60, Santa Clara, CA, USA) at a wavelength of 300–800 nm was used to identify the produced AgNPs. Morphologies, surface distributions, powder crystal structures, and dimensions of NPs were determined using X-ray diffractometer (XRD, Rad B-DMAX II, Rigacu, Tokyo, Japan), electron dispersive X-ray (EDX, Quanta, FEG240, Hillsboro, OR, USA), field emission scanning electron microscopy (FE-SEM, Quanta, FEG240, USA), transmission electron microscopy (TEM, JEM-1010, JEOL, Peabody, MA, USA), and zetasizer (Malvern, Mastersizer 3000, Malvern, UK). The surface topology of the produced AgNPs was revealed via atomic force microscopy (AFM, Park NX10, Suwon, Republic of Korea) analysis. Fourier transform infrared (FT-IR, Agilent, Cary 630, Santa Clara, CA, USA) analysis was utilized to determine the groups liable for a reduction in the extracted material. The steadiness and enduringness of the produced nanomaterials were ascertained via a thermogravimetric/differential thermal analyzer (TGA-DTA, SDT-Q600, TA Instrument, New Castle, DE, USA).

### 3.5. Antipathogenic Activity of Silver Nanoparticles

The modified minimum inhibitory concentration (MIC) approach was utilized to assess the efficiency of PR-AgNPs against pathogenic microorganisms [15]. The n96-well microplates were filled with Roswell Park Memorial Institute (RPMI 1640, Sigma-Aldrich, Darmstadt, Germany) medium for yeast and Mueller Hinton Broth (M-H Broth, Sigma-Aldrich, Darmstadt, Germany) medium for bacterial strains. The solution containing AgNPs and bacteria was supplemented to the medium. The medium was incubated for one day. To get the MIC value, the concentration in the well where the growth first appeared was measured. Fluconazole, colistin, and vancomycin standard antibiotics (128 mg mL^−1^) and 40 mM AgNO_3_ solution were used to assess the antipathogenic efficiency of PR-AgNPs.

### 3.6. Cell Culture and Cytotoxic Potentials of Silver Nanoparticles

RPMI medium was used for HUVEC and CACO-2 cell lines, and Dulbecco’s Modified Eagle medium (DMEM, Gibco, Loughborough, UK) was used for T98-G and MCF-7 lines. All the cell lines were incubated in T75 culture flasks at 37 °C in 5% CO_2_. A total of 10% fetal bovine serum (FBS), 100 U mL^−1^ penicillin, and 100 U mL^−1^ streptomycin were supplemented into both mediums. When the cells reached 80–90% confluence, they were taken from the flasks, and their numbers were determined using the hemocytometric method. The counted cells were inoculated into 96-well plates in 90 µL of the medium, with 7.5 × 10^3^ cells for HUVEC, 8 × 10^3^ cells for CACO-2, 5 × 10^3^ cells for MCF-7, and T98-G in each well. The cell inoculations were performed in triplicate and two plates (to perform two different time treatments, 24 and 48 h). The AgNPs synthesized at diverse concentrations (500, 250, 125, 62.5, 31.25, and 15.625 µg mL^−1^) were applied to the plates that were inoculated the next day. Ultrapure water was applied to the cells in the control group. 3-(4,5-dimetiltiazol-2-il)-2,5-difeniltetrazolium bromid (MTT) test was conducted to ascertain the changes in the cell viability at 24 and 48 h after the application [41].

In each well, 10 µL of 5 mg mL^−1^ MTT solution was added, and the cells were incubated for three hours at 37 °C in an environment with 5% CO_2_. After three hours, 100 µL of dimethyl sulfoxide (DMSO) replaced the medium. The plates were placed on a plate shaker at room temperature for 20 min, and the optical density (OD) of the wells was measured at a wavelength of 540 nm using a plate reader (Multiscan Go, Thermofisher, Waltham, MA, USA). The experiments were performed three times to ensure accuracy [42].

The cytotoxic activity studies were performed at the Dicle University Veterinary Faculty Cell Culture Laboratory (Diyarbakir, Turkey).

### 3.7. Statistically Analyze

Statistical analysis of the data obtained from the study was made using the SPSS pocket program (IBM, Version 21.0, Armonk, NY, USA).

## 4. Conclusions

This study discovered that AgNPs can be manufactured from the leaves of the *Papaver rhoeas* plant, which is widely distributed in Anatolia, with an easy, low-cost, and environmentally friendly method. UV-vis, EDX, FE-SEM, TEM, XRD, AFM, FT-IR, TGA-DTA, and zetasizer were used to conduct structural and thermal characterization experiments. It was recognized that the produced nanomaterial had a homogeneous distribution and was mostly spherical. In comparison to the other species, *Candida albicans* were shown to be more sensitive to the biocidal effects of PR-AgNPs. The AgNPs used in this study were found to have inhibitory effects on the growth of CACO-2, MCF-7, and T98-G cancer cells. The death rate of these cancer cells also increased with increasing concentration of the AgNPs, regardless of the exposure time. However, it was observed that these same AgNPs caused toxicity in healthy HUVEC cells. The produced biogenic, affordable, and biocompatible AgNPs are considered suitable for antibacterial applications in both human and veterinary medicine, including drug delivery and wound dressing. However, further research is needed to better evaluate the potential of these AgNPs for use in nanomedicine and nanochemistry, which may require extensive in vivo testing.

## Figures and Tables

**Table 1:** Minimum inhibitory concentration (MIC) outcomes (mg L^−1^) of *Papaver rhoeas*-leaf extract-based silver nanoparticles (PR-AgNPs), silver nitrate (AgNO_3_) solution, and common antibiotics.

Pathogen Microorganisms	PR-AgNPs	AgNO_3_ Solution	Control Antibiotics *
*Escherichia coli*	0.750 ± 0.10	0.66 ± 0.10	2.00 ± 0.10
*Staphylococcus aureus*	1.500 ± 0.15	2.65 ± 0.20	2.00 ± 0.15
*Pseudomonas aeruginosa*	6.000 ± 0.20	0.66 ± 0.10	2.00 ± 0.20
*Bacillus subtilis*	3.000 ± 0.15	1.32 ± 0.10	1.00 ± 0.15
*Candida albicans*	0.375 ± 0.10	0.66 ± 0.15	2.00 ± 0.10

* Vancomycin was used for Gram-negative bacteria, colistin was used for Gram-positive bacteria, and fluconazole was used for *C. albicans*. All the treatments were performed in triplicate. Means and standard deviations were reported as mean ± SD.

## Data Availability

All data used to support the findings of this work are included in the article.

## Sample Availability

Samples of the compounds are available from the corresponding authors.
